# Glycosuria and Allied Conditions

**Published:** 1913-06

**Authors:** 


### Reviews of Books.

**Glycosuria and Allied Conditions.** By P. J. CAMMIDGE, M.D. Pp. vii. 467. London: Edward Arnold. 1913. 16s. net.

The senior student and practitioner who is interested in diabetes and allied conditions will find a large amount of information in Dr. Cammidge's book, which we can thoroughly recommend as a painstaking and interesting monograph on this very complicated subject. Naturally a large portion of the work is taken up with questions of physiological chemistry and metabolism, but there is also a good deal of clinical description and not a few detailed cases, illustrating points brought forward by the author.

The amount of space devoted to the purely chemical and physiological chapters, though adding to the completeness of the author's treatment of the subject, might, we think, have been considerably reduced. The greater part of the material can be easily found in other books, and the space gained by this compression could well have been utilised in expanding the chapters devoted to diabetes and glycosuria, which suffer in places from a too brief and condensed method of treatment. The chapter on experimental glycosuria is a very adequate one, and in some ways superior to those which follow. It may be read with great advantage by students who are going up for the higher examinations in medicine. In the chapter on transitory glycosuria, there seems to be a little confusion as to the headings of sections, which somewhat obscures the arrangement of the matter. The clinical distinction, however, between purely transitory glycosuria and those cases in which the symptom returns whenever special strain is put on the sugar metabolism is well brought out, and illustrated by some interesting cases. In the next chapter the account of the changes in the urine and blood in "true diabetes" is very fully given, but the symptomatology and complications are described in a rather perfunctory manner, and the author is tempted, as in the case of acetonuria, to digress somewhat considerably from the clinical aspect of the condition. The next chapter, on the pathology and diagnosis, suffers somewhat from condensation. Moreover, the author has not been able to avoid a partial repetition of some matters discussed in the chapter on experimental glycosuria. The diagnosis deals very largely with the question of pancreatic involvement and the various methods for determining the functional efficiency and organic soundness of the pancreas, on which the author is an authority. The chapter on metabolism begins with an account of normal metabolism, which is perhaps rather a luxury than a necessity in a work of this sort. The special metabolic anomalies of diabetes are rather briefly dealt with, but the matter on the whole is stated as clearly as its difficult character will allow. The ninth chapter, that on treatment, contains a number of valuable tables and charts which should prove of considerable practical use to those who wish to undertake the scientific as opposed to the haphazard treatment of their cases. It emphasises the need of individual tratment, and outlines a system based on a determination of the clinical data of the case. It is well worthy of careful consideration by all practitioners who can obtain these data and act upon them, though it is characterised rather by scientific precision than by clinical insight. The remaining chapters deal with subjects of somewhat academic interest, which are presented in an adequate manner, but not more fully perhaps than is usual in the larger "systems" of medicine. There are useful bibliographies at the end of each chapter and an index of nine pages.

The work as a whole is to be commended as a useful *résumé* of the present position of our knowledge and also as an interesting account of the author's special experience from the chemical standpoint. We hope that when a new edition is required more care will be taken in revising the proofs, and that the author will definitely make up his mind on certain points in orthography. Not only is he in doubt as to the final "e" in such words as "glycerine" and "formaldehyde," but we also find "sterio isomer," "mutarotation," "anærobic" and "guiacol." Phlorhizin throughout is spelt "phloridzin," which seems a pity since attention has recently been called to this common error. Fractions of a gram are frequently coupled with a plural substantive, and there are numerous misprints of a more or less obvious character. These, however, are minor blemishes, and we congratulate Dr. Cammidge on the result of his work and the publisher on a very useful and well-set-up volume.

